# The effects of minimum unit pricing for alcohol on food purchases: Evaluation of a natural experiment

**DOI:** 10.1016/j.ssmph.2022.101174

**Published:** 2022-07-19

**Authors:** Daniel Kopasker, Stephen Whybrow, Lynda McKenzie, Paul McNamee, Anne Ludbrook

**Affiliations:** a University of Glasgow, SPHSU, United Kingdom; b University of Aberdeen, HERU, United Kingdom; c University of Aberdeen, Rowett Institute, United Kingdom

## Abstract

**Background:**

On the 1st of May 2018 Scotland became the first country to introduce minimum unit pricing (MUP) for alcohol sales. The objective of this study is to identify the effects of this policy instrument on food purchasing by evaluating a natural experiment.

**Methods:**

Longitudinal analysis compares regions with similar characteristics but differing exposure to MUP (Scotland and the north of England). Secondary data from the Kantar Worldpanel on itemised purchases between April 2017 and April 2019 provided a total sample of 8051 households. The outcomes analysed are weekly household expenditure (£s) and purchase volume (grams), both overall and disaggregated to 16 product categories.

**Results:**

Following the introduction of MUP, total household food expenditure in Scotland declined by 1.0%, 95%CI [-1.9%, −0.0%], and total food volume declined by 0.8%, 95%CI [-1.7%, 0.2%] compared to the north of England. There is variation in response between product categories, with less spending on fruit and vegetables and increased spending on crisps and snacks.

**Conclusion:**

Minimum unit pricing for alcohol has displaced some household food purchasing and the pattern of changes in food categories appears to be less desirable from a healthy diet perspective. However, changes caused by a minimum price at a nominal 50 pence per unit of alcohol are relatively small.

## Highlights

• Minimum unit pricing for alcohol has displaced some household food purchasing.
• The pattern of changes is less desirable from a healthy diet perspective.
• Changes are relatively small at the current level of minimum unit price.

## 1 Introduction

On the 1^st^ of May 2018 Scotland became the first country to introduce minimum unit pricing (MUP) for alcohol sales as a way to reduce alcohol misuse and its negative consequences. Legislation required that the effect of the intervention should be independently reviewed after 5 years. A programme of research to measure the consequences of this intervention is being co-ordinated by Public Health Scotland (Beeston et al., 2020) and includes the evaluation of effects on household expenditure.

The mechanism underpinning the design of the MUP policy instrument is relatively straightforward. An increase in the minimum price per unit of alcohol will reduce the amount of alcohol purchased, all other things being equal. Modelling conducted prior to the introduction of MUP (Angus et al., 2016) predicted that, on average, households will buy less but spend more on alcohol. This is due to the reduction in quantity purchased being less than proportionate to the price increase. The model estimated an increase in the monetary value of total purchases of alcohol of 0.7% (£5 per drinker per year) based on a minimum price of 50p per unit (10ml) of alcohol.^2^ Emerging evidence following the introduction of MUP has been consistent with a somewhat larger expenditure effect than predicted by prior modelling, although expenditure effects have either had a wide confidence interval (O'Donnell et al., 2019) or only been implied by a closely related measure, such as price per unit of alcohol purchased (Anderson et al., 2021; Xhurxhi, 2020). Where budgets are fixed and constrained, an increase in household expenditure on alcohol resulting from MUP would reduce the budget available for other goods and services and so displace other purchases.

Due to the novel nature of the MUP policy, little is known of the extent to which increased alcohol expenditure will displace other purchases. However, a complementary relationship between alcohol and food intake has been identified in a number of studies (Breslow et al., 2010, 2013; Grech et al., 2017; Kwok et al., 2019) and is confirmed by a recent review (Fong et al., 2021). This indicates that a reduction in alcohol intake is predicted to be accompanied by a reduction in food intake. A recent economic study, using UK data, also indicates that a price increase for alcohol is predicted to decrease both alcohol and food consumption (Moore et al., 2021). The existing literature does not however estimate the size of the effect of a specific price intervention, such as MUP, and none of the studies examined purchasing behaviour. This study aims to address this knowledge gap by testing for the existence of potential unintended effects on food purchasing. The analysis uses household purchase microdata, collected by Kantar Worldpanel (KWP), to compare household food and non-alcoholic drink purchases (shortened to food purchases hereafter) before and after MUP. The main hypothesis to be tested is that a change in household food purchases occurred in Scotland following the introduction of MUP for alcohol.

## 2 Methods

The introduction of MUP for alcohol by the devolved government in Scotland, without equivalent legislation in England, creates the conditions for a natural experiment. A counterfactual for household purchases in the absence of MUP is provided by comparison with households in the north of England. The north of England has been chosen as the comparator group, rather than the whole of England, as the area is more similar to Scotland in terms of demography, drinking patterns and culture (Robinson et al., 2015), and for consistency with other studies of MUP being undertaken within the NHS Health Scotland (now Public Health Scotland) evaluation plan.

The analysis evaluates the natural experiment within a difference-in-differences (DID) framework. DID mimics a randomised control trial in a non-randomised setting when using secondary data and enables causal inferences to be made. Weekly food purchases by households in Scotland (the treatment group) are compared to purchases by equivalent households in the north of England (the comparator group). Both groups are observed for 53 weeks prior to the introduction of MUP in Scotland, and for 54 weeks after the policy was introduced in Scotland only^1^. The sample period covers the week ending the April 30, 2017 to week ending the May 12, 2019. The treatment period commences one day prior to the introduction of MUP on Monday the April 30, 2018 (the treatment starts one day prior to MUP implementation to enable compatibility with week dates in the KWP data).

Household purchases microdata, collected by KWP, was used to compare household food purchasing before and after MUP. In the KWP, panel members scan and report all purchases, including some non-food purchases, brought into the home, as well as providing till receipts which verify the purchases made and provide price information. Non-bar-coded items that are sold loose (for example, some fruit and vegetables) are also recorded. The dataset covers all types of outlet where purchases of food and drink to bring home can be made. This includes supermarkets, corner shops and online purchases. All expenditure was indexed by the authors to 2018 real values using the ONS annual index D7BT.

The effect of MUP on total weekly household food spending and volume of food purchased are initially analysed. Thereafter, total food purchasing is disaggregated to analyse the composition of purchasing in distinct product categories. Non-participants in product markets - households with zero purchases in all weeks for that category - are omitted from the analysis for that specific product category due to an absence of variation in the outcome variable.

The KWP data also includes data on household characteristics that are used in the analyses. Household location is used to determine exposure to MUP using the categories available in KWP. The treatment group is households with a Scottish Neighbourhood Statistics Data Zone identifier (Scottish Government, 2004). The comparator group is households with a postcode within the TV Broadcasting Audience Research Board areas for the north of England (Border England, North East, North West, and Yorkshire) (Sky Media, 2016). Households remain in the same group (treatment or comparator) throughout the sample period and those moving out of or into a treatment area (n = 38) are excluded from the analysis.

Further socioeconomic characteristics collected by KWP are used to ensure comparability between the treatment groups. The sample is restricted to households with full socioeconomic data and with at least one observation week in both the pre-MUP and post-MUP periods. Inadequate data to enable weighting (n = 1759) or being observed in only one treatment period (n = 1617) resulted in the exclusion of 3376 households. An initial review of the sample characteristics indicated a high degree of comparability between the two groups (as indicated in Table 1). However, the sample size of the unweighted comparator group (n = 6064) is substantially larger than the treatment group (n = 1987). To ensure the estimates are not influenced by heterogeneity in observable characteristics, while fully utilising all the available information, the data are pre-processed to form sample weights which match the samples using entropy balancing (Hainmueller, 2012). Entropy balancing minimises the entropy distance between variables for two groups by identifying a unique weight which matches moments from the distributions of individual variables. Weights are based on data in the week when a household is first observed in the pre-MUP period using the Stata user-written package ebalance (Hainmueller & Xu, 2013).

**Table 1:** Household characteristics at first observation within the sample.

	Scotland - unweighted (n = 1987)	North of England - unweighted (n = 6064)	North of England -weighted (weighted n = 1987)
Log years in panel	1.308 (1.774) [-0.984]	1.579 (1.259) [-1.300]	1.307 (1.774) [-0.984]
Main shopper: age	50.520 (194.1) [0.203]	50.100 (208.5) [0.210]	50.520 (194.1) [0.204]
Main shopper: age squared/100	27.470 (214.3) [0.721]	27.180 (227.1) [0.689]	27.470 (214.3) [0.722]
Household size	2.540 (1.605) [0.807]	2.728 (1.752) [0.766]	2.539 (1.605) [0.807]
Children dummy	0.304	0.345	0.304
Main shopper: male	0.291	0.257	0.291
Employment: 8–29 h	0.173	0.195	0.173
Employment: Full Time Education	0.006	0.003	0.006
Employment: Not working	0.116	0.118	0.116
Employment: Over 30 h	0.433	0.399	0.433
Employment: Retired	0.235	0.249	0.235
Employment: Under 8 h	0.018	0.017	0.018
Employment: Unemployed	0.019	0.019	0.019
Household income: £0 - £9999 pa	0.067	0.176	0.067
Household income: £10,000 - £19,999 pa	0.220	0.068	0.220
Household income: £20,000 - £29,999 pa	0.219	0.223	0.220
Household income: £30,000 - £39,999 pa	0.175	0.224	0.174
Household income: £40,000 - £49,999 pa	0.120	0.122	0.120
Household income: £50,000 - £59,999 pa	0.084	0.083	0.084
Household income: £60,000 - £69,999 pa	0.051	0.045	0.051
Household income: £70,000+ pa	0.064	0.059	0.064
Social class: C1	0.381	0.391	0.381
Social class: C2	0.167	0.180	0.167
Social class: D	0.153	0.134	0.153
Social class: E	0.092	0.088	0.092
Social class: AB	0.207	0.207	0.207
Household location: rural	0.207	0.138	0.207

Notes: Variance in parentheses, and skewness in brackets for continuous variables only.

The distributions of the outcome variables are characterised by many observations near the origin and substantial positive skewness. To ensure robust estimation of the model for such distributions, while also allowing for sample weights and household fixed effects, the Poisson pseudo-maximum likelihood estimator is implemented in Stata using the ppmlhdfe command (Correia et al., 2020). The model specification controls for observed time-invariant and time-varying household characteristics, unobserved time-invariant household characteristics, and seasonality in purchases. The model can be defined as:

$$Y_{hgt}=\exp\left(\beta_{1}Pop_{g}+\beta_{2}MUP_{t}+\beta_{3}D_{gt}+X_{hgt}^{\prime }\gamma +\alpha_{h}+\eta_{m}+\varepsilon_{hgt}\right)$$

where *Y* is either food spending or volume purchased aggregated over a week for household *h*, in treatment group *g*, at week *t*. The outcome variables are for either sixteen categories aggregated or a single product category. *Pop* is the treatment group dummy (1 for Scotland), *MUP* is the treatment period dummy (1 for week 54 onwards), *D* is the exposure to treatment effect (an interaction between *Pop* and *MUP*), *X* is a vector of time-varying household characteristics (age of main shopper, age of main shopper squared, household size, children in household dummy, log years in panel, spend on non-food), and time-invariant household characteristics (both observed and unobserved) are captured by the household fixed effect *α*. Within the household fixed effect, the observed time-invariant characteristics are employment status, household income, social class, and an urban/rural dummy. *η* is a dummy variable for month *m* included to allow for seasonality, and *ε* is the idiosyncratic error. Standard errors are clustered at the household level such that they are robust to arbitrary heteroskedasticity and within-household autocorrelation.

The age of the main shopper (and its square) is included within the model to control for variation in purchasing at different life stages. Household size and children in the household control for households of varying composition having different wants and needs. The time a household has been in the panel controls for possible measurement error due to respondent fatigue, a potential issue in consumer panel data (Leicester & Oldfield, 2009). Spending on non-food items is included to control for other aspects affecting household budgets.

An important assumption of the DID approach is that prior to the introduction of MUP there was a common trend in food purchases for both the treatment and the control groups that would be expected to continue in the absence of the intervention. This enables potential outcomes for the treatment group in the absence of MUP to be indicated by the post-intervention trend in the control group plus or minus any difference between the groups in the levels of the pre-intervention trends. A test of this assumption was conducted by including dummies for each month-year period and interacting these with the treatment group dummy. After estimating the model, a chi^2^ test to assess whether the sum of all pre-MUP interactions between time and the treatment group dummy was equal to zero was conducted. Failure to reject the null hypothesis would indicate that there was no statistically significant difference in the pre-MUP time trends in Scotland and the north of England.

In the main analysis of aggregate food spending and volume, households are observed and included in the sample when there is at least one non-zero purchase in at least one product category in a week (see Appendix for category list). As such, zero-purchase weeks within some, but not all, categories are included in the analysis since the household is reliably observed. Weeks with no recorded purchases across all categories, when households are not observed, are excluded from the main analysis and investigated in sensitivity analysis by imputing zeros for missing weeks in months with at least one non-zero food purchase week. The main analysis includes both households that purchase alcohol and those that do not. Although the latter will not be directly affected by MUP for alcohol, they may be indirectly affected if retailers change their pricing strategy using potential increased revenue from MUP. In sensitivity analysis, the sample is restricted to households that purchase alcohol.

## 3 Results

### 3.1 Descriptive statistics

Table 1 provides descriptive statistics demonstrating the extremely high degree of comparability between the treatment groups achieved by entropy balancing, while still utilising all the information within the larger comparator sample. The table provides both weighted and unweighted descriptive statistics. Only the comparator group is weighted. Weighting is used to strengthen the counterfactual for the analysis since we cannot observe the sample of households in Scotland had they not been exposed to MUP. Weighting provides a synthetic population that is almost identical to the Scottish sample except for being unexposed to MUP throughout the period. This strengthens the basis for inference since changes in food expenditure are more likely to be attributable to MUP than to the characteristics of the treatment and comparator groups. After weighting the north of England sample, no statistically significant differences remain between the groups. The average main shopper within a sample household is 50 years old, female, lives in a household with 2.5 people, working full time, with an annual income below £40,000. The most common household income groups are £10,000-£19,999 and £20,000-£29,999 per annum, which may be due to a substantial proportion of the sample (23.5%) being retired. It is within the lower income groups where the largest differences between the groups are observed prior to weighting, with a larger proportion of households in the north of England in the lowest income group. Likewise, a larger proportion of households in the north of England are in the £30,000 - £39,999 per annum income band than in Scotland. Weighting the north of England sample addresses these differences.

The characteristics in Table 1 have relatively low levels of time variation over the roughly two-year period analysed, either due to the nature of the characteristic, unit of measurement, or frequency of data collection by KWP. For example, average household size is 2.48 in Scotland pre-MUP and 2.47 post-MUP. The equivalent unweighted figure for the north of England is 2.68 pre-MUP and 2.65 post-MUP. Therefore, weights based on the first observation enhance comparability throughout the sample period.^2^

**Fig. 1:**
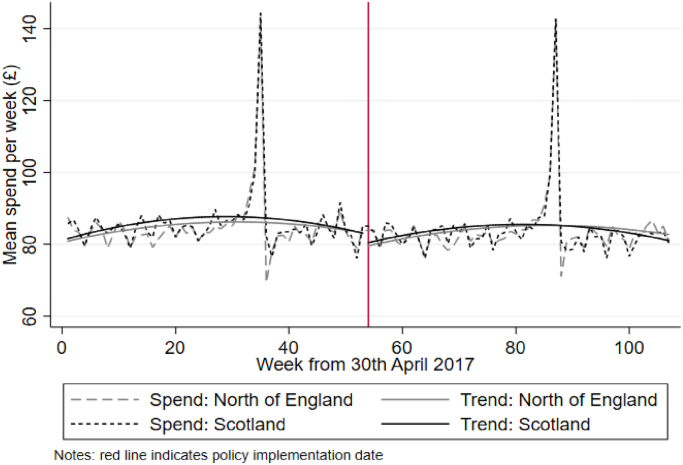
Weighted mean levels and trends in food spending per week for each sample area and period.

**Fig. 2:**
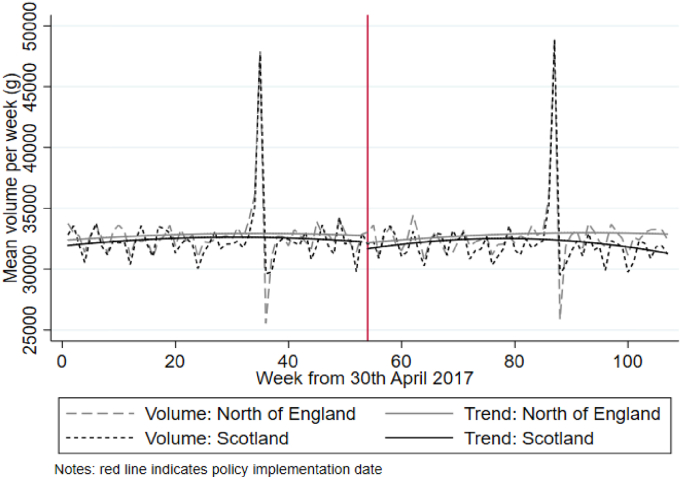
Weighted mean levels and trends in food volume per week for each sample area and period.

Fig. 1, Fig. 2 illustrate changes in the weighted mean levels and trends in food spending and volume of food purchased during the pre-MUP and post-MUP periods for both the treatment (Scotland) and comparator (north of England) groups. In the pre-MUP period trends in both outcomes appear comparable, such that visual inspection broadly supports the common trends assumption. Pre-MUP food spending is higher in Scotland than in the north of England, although there is some narrowing of the difference between the pre-MUP trends for food spending in the weeks leading up to the policy implementation date. Given that the introduction of MUP in Scotland was widely publicised and required retailers to adjust pricing, some level of anticipation is plausible. Following the introduction of MUP there are small but distinct changes in the trends. For food spending, the difference between the trends narrows then switches in the post-MUP period, such that by the end of the sample period mean food spending is higher in the north of England than in Scotland. For food volume, the difference between the trends widens from November 2018 onwards (approximately week 80) as the post-MUP period progresses, with mean food volume higher in the north of England than in Scotland.

Table 2 provides descriptive statistics for total food spending and total volume of food purchased. Descriptive statistics disaggregated to the product category level are available within the Appendix (Table A1). From Table 2 it is clear that prior to the introduction of MUP in Scotland there were statistically significant differences between the treatment groups in both food spending and volume purchased. At the mean, food spending was 107 pence higher, and volume was 367g lower, per week in Scotland compared to the north of England. Although Table 2 indicates a pre-MUP difference in the levels of food purchases, tests of trends in food purchases fail to reject that Scotland and the north of England had a common trend in total weekly food spending (chi^2^ = 2.61, p = 0.106) and total weekly volume of food purchased (chi^2^ = 0.62, p = 0.432). The output from the tests of common trends, plus additional tests of common trends for a linear model, and a summary of category level tests are included in the Appendix (Table A19 and Table A20).

**Table 2:** Descriptive statistics for total food spending and total food volume per week for each sample area and period.

	Scotland	North of England	*t*-test of equality	Scotland	North of England	*t*-test of equality
	Spend (£)	Spend (£)		Volume (g)	Volume (g)	
Pre-MUP	85.91	84.84	3.96 (p < 0.01)	32462	32829	3.86 (p < 0.01)
Post-MUP	83.94	83.80	0.55 (p > 0.1)	32182	32801	6.59 (p < 0.01)
*t*-test of equality	6.09 (p < 0.01)	5.27 (p < 0.01)		2.47 (p < 0.05)	0.40 (p > 0.1)	

Note: Two-way *t*-test of equality at weighted means.

In the second period in Table 2, following the introduction of MUP on the 1^st^ of May 2018, food spending and volume purchased decreased in both Scotland and the north of England. However, the change was more substantial in Scotland. Mean food spending fell by almost £2 per week in Scotland compared to just over £1 per week in the north of England. The mean volume of food purchased fell by 280g per week in Scotland compared to only 28g per week in the north of England. In the north of England, the change in volume purchased between treatment periods is not statistically significant at conventional levels. The descriptive statistics in Table 2 indicate that the difference between the two treatment groups increased for food volume following the introduction of MUP in Scotland but narrowed for food spending.

### 3.2 Total food spending and purchase volume

Table 3 reports the results of Poisson pseudo-maximum likelihood regression models for the main outcome variables^3^. Food spending in Scotland was 1.0%, 95%CI [−1.9%, −0.0%] lower compared to the north of England following the introduction of MUP for alcohol, a change of approximately 86 pence per week at the mean. Likewise, the volume of food purchased in Scotland was 0.8%, 95%CI [−1.7%, 0.2%] lower, although not statistically significant (p > 0.1). This decrease in volume is equivalent to approximately 260g per week at the mean.

**Table 3:** Percentage change per week in total household food and non-alcoholic drink purchases following the introduction of minimum unit pricing for alcohol in Scotland.

	Spending (%)	Volume (%)
Post-MUP period	0.5 [-0.7,1.6]	1.8*** [0.7,2.8]
Treatment effect (Post MUP period in Scotland)	−1.0** [-1.9,-0.0]	−0.8 [-1.7,0.2]
Age of shopper	5.0*** [3.2,6.8]	4.0*** [2.3,5.7]
Age of shopper squared/100	−5.0*** [-6.3,-3.7]	−4.3*** [-5.6,-3.1]
Total people in household	5.9*** [4.3,7.5]	5.6*** [4.2,7.0]
Children dummy	−5.0** [-9.6,-0.4]	−2.9 [-7.6,1.9]
Log years in panel	−5.1*** [-6.9,-3.2]	−5.3*** [-7.1,-3.5]
Spend: Non-food	1.0*** [1.0,1.1]	1.0*** [0.9,1.1]
Month of purchase: reference January		
Month of purchase: February	2.3*** [1.5,3.0]	1.4*** [0.7,2.0]
Month of purchase: March	4.1*** [3.3,4.8]	2.0*** [1.3,2.7]
Month of purchase: April	3.0*** [2.2,3.8]	1.6*** [0.9,2.3]
Month of purchase: May	2.4*** [1.3,3.5]	1.1** [0.1,2.0]
Month of purchase: June	1.5** [0.2,2.7]	0.1 [-1.0,1.2]
Month of purchase: July	1.0 [-0.3,2.2]	0.4 [-0.7,1.5]
Month of purchase: August	1.9*** [0.7,3.2]	0.7 [-0.4,1.8]
Month of purchase: September	1.5** [0.2,2.8]	−0.8 [-2.0,0.4]
Month of purchase: October	2.3*** [1.0,3.5]	−0.9 [-2.1,0.2]
Month of purchase: November	4.4*** [3.1,5.7]	−0.1 [-1.3,1.1]
Month of purchase: December	22.1*** [20.7,23.5]	10.8*** [9.5,12.0]
Observations	687059	687059
Households	8051	8051
Pseudo-R^2^	0.407	0.487

95% confidence intervals in brackets (using standard errors clustered at household level).

Percentage changes are approximate (see footnote c) *p < 0.10, **p < 0.05, ***p < 0.01.

Table 3 indicates that food expenditure increases throughout the year, with spending in December approximately 22% higher per week than in January. Food expenditure increases with the age of the main shopper, but at a declining rate. A one person increase in household size is associated with an increase in food spending of 5.9% per week, although the children dummy variable indicates that if a household changes from having no children to children, this expenditure increase from household size is lower. We also find evidence of respondent fatigue with reported household expenditure decreasing with time spent in the KWP, a feature common to consumer microdata (Leicester & Oldfield, 2009). The model also indicates that spending on food increases with spending on non-food products. The results for food volume show a broadly similar pattern, although the effect sizes are often slightly smaller.

### 3.3 Product category spending and volume purchased

While Table 3 indicates a reduction in household food purchases in Scotland following the introduction of MUP for alcohol, the potential for this reduction to have health consequences is dependent on the categories of food that have changed. Table 4 reports estimates of the main treatment effect on food spending and volume purchased by product category.

**Table 4:** Percentage Change Per Week in Food and Non-alcoholic Drink Category Spending and Purchase Volume Following the Introduction of Minimum Unit Pricing for Alcohol in Scotland (Treatment effect coefficient Only).

	Spend (%)		Volume (%)	
Category:				
Canned food	1.0	[-1.9,4.0]	0.5	[-2.1,3.1]
Convenience food	−0.6	[-2.3,1.2]	0.2	[-1.5,2.0]
Rice and pasta	−1.6	[-4.8,1.6]	0.8	[-2.2,3.7]
Dairy	−1.4**	[-2.7,-0.2]	−1.3*	[-2.8,0.1]
Fish	−1.4	[-5.1,2.4]	−4.1**	[-7.5,-0.6]
Meat	−1.2	[-3.3,0.8]	−0.2	[-2.1,1.8]
Cereal	−3.5***	[-6.0,-1.0]	−2.0*	[-4.4,0.3]
Fruit and veg	−2.5***	[-4.3,-0.8]	−1.2	[-2.8,0.3]
Tea and coffee	0.7	[-2.6,3.9]	2.2	[-0.6,5.0]
Juice	−1.2	[-6.2,3.8]	−2.4	[-7.4,2.6]
Home cooking	−1.7	[-4.0,0.6]	−0.8	[-3.1,1.6]
Biscuits and bakery	−0.5	[-2.0,1.1]	0.1	[-1.3,1.5]
Crisps and snacks	2.5**	[0.2,4.9]	2.0*	[-0.3,4.4]
Soft drinks	−1.8	[-4.7,1.1]	−2.5*	[-5.1,0.2]
Confectionery	0.5	[-1.7,2.6]	1.4	[-1.3,4.2]
Slimming	−18.0	[-75.0,39.0]	−46.0	[-101.1,9.1]

95% confidence intervals in brackets (using standard errors clustered at household level).

Category-level models include age of shopper, age of shopper squared/100, total people in household, children dummy, log years in panel, non-food spend in week, and month of purchase. Full regression output in supplementary Appendix.

Common trends assumption violated (see Table A20) for tea and coffee spend (p = 0.042), and fish volume (p = 0.045).

Percentage changes are approximate (see footnote c). *p < 0.10, **p < 0.05, ***p < 0.01.

Statistically significant reductions (p < 0.05) in spending following the introduction of MUP in Scotland are observed for dairy, cereals, and fruit and vegetables. A statistically significant increase is observed for crisps and snacks only (p < 0.05). However, each of the changes are relatively small. In monetary terms at the mean, the largest observed change is a 2.5%, 95%CI [−4.3%, −0.8%] reduction in the £6.30 spent per week by households on fruit and vegetables, roughly equivalent to 16 pence or the cost of a single banana (see Table A1 for mean expenditure by product category).

### 3.4 Sensitivity analysis

In sensitivity analysis the sample was restricted to households that purchase alcohol. This did not significantly alter the main results with the estimate for food spending being a 0.9% reduction, 95% CI [−1.9%, −0.0%] and for food volume a reduction of 0.8%, 95%CI [−1.7%, 0.2%]. Separately, the imputation of zero purchase weeks within months where the household was known to be active within the panel increased the magnitude and statistical significance of post-MUP effects for both food spending and volume of food purchased. The estimate for food spending in this sensitivity analysis is a reduction of 1.5%, 95%CI [−2.6%, −0.4%]. For food volume the estimate is a reduction of 1.2%, 95%CI [−2.3%, −0.8%]. As such, the main results presented in Table 3 are robust and relatively conservative.

To test for policy anticipation, we first split the twelve weeks prior to MUP implementation into two six-week periods and repeated our main analysis. This indicated statistically significant reductions in food spending in Scotland compared to the north of England in the six weeks immediately prior to MUP implementation, but not in the six weeks prior to that (see Table A21). No statistically significant evidence of anticipation was observed for food volume. We additionally repeated our analysis with the policy implementation date starting six weeks earlier on Monday March 19, 2018. This has the effect of increasing the size of the percentage change in both food spending and volume due to the implementation of the policy (see Table A22). While the absence of anticipation is one assumption of DID analysis, this assumption may be unrealistic for a widely publicised policy affecting the whole population of Scotland. Violation of this assumption has the effect of understating the reduction in food spending in Scotland since the introduction of MUP.

Implementing a linear fixed effects model, rather than the Poisson pseudo-maximum likelihood estimator that was preferred due to the distribution of the outcome variables, results in slightly smaller estimates lacking statistical significance: a 76 pence (0.9%) reduction for food expenditure, 95%CI [-£1.54, £0.33], and a 220g (0.7%) reduction in food volume, 95%CI [−522g, 83g]. This indicates that the use of an estimation method that is more appropriate to the distribution of outcomes resulted in greater precision in the estimates, in addition to providing stronger support for the common trends assumption, as stated earlier.

An unweighted analysis resulted in effect estimates of a similar magnitude to those in Table 3 but lacking statistical significance. The estimates were a reduction of 0.8%, 95%CI [−1.7%, 0.2%], for food spending, and a reduction of 0.6%, 95%CI [−1.5%, 0.3%], for food volume. This indicates that the use of weights to improve comparability between the groups resulted in greater precision in the estimates.

## 4 Discussion

The results of this analysis indicate that MUP for alcohol in Scotland caused a small, statistically significant, reduction in household food spending (1.0%). There is a smaller, non-significant reduction in the volume of food purchased (0.8%). The reduction in food spending has not been spread equally across food categories, and the pattern of changes in food categories appears to be less desirable from a healthy diet perspective, with lower spending on fruit and vegetables, and increased spending on crisps and snacks than would otherwise have been expected. However, there was no significant change in the volume of fruit and vegetables purchased.

A differing pattern of responses in spending and volume purchased would be consistent with consumer responses to larger economic shocks, such as the 2008-9 recession, when food and drink expenditure increased by a smaller percentage than the price increase, with a mixture of buying less and ‘trading down’ i.e. buying a cheaper product of the same type (Department for Environment Food and Rural Affairs, 2012). Whilst MUP provides a smaller shock to the consumer budget, we were exploring a similar phenomenon in considering both food spending and quantity purchased.

A further factor might be competition in the food and drink retail sector and the use of price discounts to drive footfall. MUP for alcohol would limit the scope for reduced price offers on alcohol but could potentially increase such offers on food products. Prior to MUP being introduced, modelling estimated an increase in revenue to retailers of 9.6%/£41m (Angus et al., 2016). Less healthy foods are promoted more often, with crisps and snacks having one of the highest levels of promotion (Food Standards Scotland, 2018).

The key pathway being investigated is that increased spending on alcohol in Scotland following the introduction of MUP will displace some food expenditure within constrained household budgets. An annual reduction in household food expenditure of £45 (1.0% per week at the mean) is larger than the increase in annual consumer spending on alcohol of £5 per drinker predicted by modelling prior to the introduction of MUP (Angus et al., 2016). However, in monetary terms, the confidence interval in Table 3 has substantial overlap with empirical evidence showing MUP to have increased household alcohol expenditure in Scotland by 61p per week, equivalent to £32 per annum (O'Donnell et al., 2019). Therefore, our results are consistent with an emerging pattern of evidence from other evaluations of MUP in Scotland.

Early evidence indicates that MUP has reduced the average volume of off-trade alcohol purchased within Scotland by 3.5% (Giles et al., 2021). Consequently, it would be expected that the policy has positive direct effects on population health. Unintended consequences of MUP, such as unhealthy changes to the pattern of food spending, may negate some of the positive health changes achieved by the policy. However, the identification of a statistically significant reduction in food spending, and the pattern of changes, does not automatically imply substantial health consequences. When considering potential health consequences it is important to make a distinction between the pattern and the scale of changes.

Although the results presented in this paper indicate potentially unhealthy changes, there are factors that could limit concern: the effects are relatively small in percentage terms, and in some food categories the changes are one component of an overall increase in the absolute level. The largest monetary change identified in the results was a 16 pence reduction in the volume of fruit and vegetables purchased (see Table 4 and Table A1). The largest and most statistically significant volume reduction observed, 4.1% for fish, is equivalent to around 7g less per household per week at the mean. Likewise, the increase in volume for crisps and snacks of 2.0% is equivalent to around 5g more per household per week. In addition, a common pattern is that expenditure reductions are not fully reflected in volume reductions. Further analysis is currently being conducted to estimate the overall net nutritional and health impacts.

Most of the statistically significant changes identified in the analysis indicate lower food spending in Scotland compared to the north of England following the introduction of MUP. However, this does not always indicate lower levels of food spending within Scotland post-MUP compared to Scotland pre-MUP. The overall effect for Scotland is a combination of the post-MUP change that is common to Scotland and the north of England, and the relative change in Scotland post MUP. In the case of fruit and vegetables, the relative post-MUP reduction of 2.5% reported in Table 4 must be combined with a common post-MUP increase of 5.8% (see Table A10 in Appendix). Therefore, it is likely that fruit and vegetable expenditure increased overall in Scotland post-MUP, but the introduction of MUP restricted the level of this increase.

One limitation of this study is that on-sales alcohol expenditure is not captured by the KWP dataset. MUP covers all alcohol sales in Scotland. On-sales generally had a price per unit above the MUP prior to the introduction of the policy. Some modest diversion of spending towards on sales alcohol, due to a small change in relative price, was allowed for in earlier modelling (Angus et al., 2016). However, data suggests that on-sales of alcohol were stable (in terms of litres of pure alcohol per drinker) in Scotland during 2018 and 2019, decreasing slightly from the 2017 level (Giles et al., 2021). Therefore, there is no evidence that substitution between on-sales and off-sales would influence our result that MUP caused a reduction in food purchases in Scotland.

## 5 Conclusion

Minimum unit pricing for alcohol has displaced some household food purchasing and the pattern of changes in food categories appears less desirable from a healthy diet perspective. The small reduction in total food spending identified here resulted from a policy with the minimum set at a nominal 50 pence per unit of alcohol. It is possible that a higher minimum price would result in larger reductions in weekly food spending. If these followed a similar pattern of reduced spending on positive health producing foods, and greater spending on crisps and snacks, the potential for unintended negative health outcomes would also increase. As MUP is not index linked, the real value of the minimum unit price is reducing over time due to inflation. Therefore, as more data emerges it will be possible to assess the effects of MUP at different real values to identify the consequences of marginal changes in the level of MUP. Policymakers should therefore continue to remain cognisant of potential displacement effects from MUP to food, given that the evidence presented here suggests that the current level of MUP affected food purchasing decisions.

## Data sharing

Kantar Worldpanel data are not publicly available but can be purchased from Kantar Worldpanel (http://www.kantarworldpanel.com). The authors are not legally permitted to share the data used for this study.

## Funding

The Health Economics Research Unit is supported by the 10.13039/501100000589Chief Scientist Office (CSO) of the Scottish Government Health and Social Care Directorates (SGHSC). Financial support for the study was received from the 10.13039/100014589CSO. DK was also funded by the 10.13039/501100000265Medical Research Council (MC_UU_00022/2) and the 10.13039/100012095Scottish Government Chief Scientist Office (SPHSU17). The views expressed here are those of the Health Economics Research Unit (10.13039/501100000882University of Aberdeen) and not necessarily those of any funding body. Results and interpretation are the authors' own from Kantar Worldpanel data.

## Ethics statement

This project used anonymised secondary data for which informed consent was received by the original data collectors. No further ethical approval was required for this project.

## Contributors

AL and PM conceived the study. AL, PM, and DK defined the analytical strategy. DK and SW performed statistical analyses and provided preliminary interpretation of findings. DK drafted the manuscript. AL, PM, SW, LM, and DK critically interpreted the results, revised the manuscript, provided relevant intellectual input, and read and approved the final manuscript. The corresponding author attests that all listed authors meet authorship criteria and that no others meeting the criteria have been omitted.

## Declaration of interests

The authors declare that they have no known competing financial interests or personal relationships that could have appeared to influence the work reported in this paper.

## Data Availability

The authors do not have permission to share data.

## Footnotes

1 The length of the pre-MUP and post-MUP observation periods differ slightly so as to fully utilise all the data available. The observations periods are identical for both the treatment and comparator group.

2 One further variable, spending on non-food items, is included within the model specification but is not included in Table 1. The reason for this is that this variable has substantial variation within households between weekly data points, so any arbitrary comparison point is unlikely to be representative of other periods. This variable has an unweighted mean (standard deviation) of £8.64 (£11.84) for Scotland and £8.45 (£11.15) for the north of England in the pre-MUP period.

3 To obtain fully accurate estimates of changes, coefficients should be transformed using their exponent and then deducting one. However, when coefficients are close to zero, the untransformed coefficients provide almost identical estimates after rounding percentages to one decimal place. For consistency with the tables of results in the Appendix, while not altering the interpretation, untransformed coefficients are used throughout the results and discussion sections.
