# Supplementary material for: The effects of minimum unit pricing for alcohol on food purchases: Evaluation of a natural experiment
Source: SSM Popul Health. 2022 Jul 19;19:101174. doi: 10.1016/j.ssmph.2022.101174 (PMC9310105; doi:10.1016/j.ssmph.2022.101174)
Supplement: Multimedia component 1 [file mmc1.docx]

# **Appendix**

| Food and non-alcoholic drink product categories: | Kantar Worldpanel Market Sector(s) |
| --- | --- |
| Canned food | Canned goods |
| Convenience food | Chilled convenience, frozen prepared foods |
| Rice & pasta | Savoury carbohydrates and snacks |
| Dairy | Dairy products |
| Fish | Fresh fish, frozen fish |
| Meat | Fresh meat, fresh poultry and game, frozen meat, frozen poultry and game |
| Cereal | Packet breakfast |
| Fruit & veg | Fruit and vegetables and salads |
| Tea & coffee | Hot beverages |
| Juice | Chilled drinks |
| Home cooking & condiments | Pickle and table sauce and condiments, savoury home cooking, sweet home cooking |
| Biscuits & bakery | Ambient bakery products, biscuits, chilled bakery products |
| Crisps & snacks | Take home savouries |
| Soft drinks | Take home soft drinks |
| Confectionery | Frozen confectionery, take home confectionery |
| Slimming | Ambient slimming products |
|  |  |
| Other product categories |  |
| Non-food | Bathroom toiletries, haircare, healthcare, oral-care, other toiletries, pet care, household and cleaning products |

**Table A1. Descriptive Statistics for Product Category Dependent Variables per Week pre-MUP for Each Sample Area.**

|  | Scotland: pre-MUP | | North England: pre-MUP | |
| --- | --- | --- | --- | --- |
|  | Spend (£) | Volume (g) | Spend (£) | Volume (g) |
| Canned food | 2.01 (3.81) | 1081.54 (1893.59) | 2.27 (3.97) | 1236.02 (1999.23) |
| Convenience food | 17.74 (22.27) | 3797.41 (4528.72) | 18.07 (22.61) | 4046.68 (4812.98) |
| Rice and pasta | 1.08 (2.21) | 417.51 (959.2) | 1.05 (2.32) | 419.59 (1111.71) |
| Dairy | 9.44 (8.48) | 7220.49 (6608.92) | 9.65 (8.34) | 7912.3 (7281.77) |
| Fish | 1.86 (4.93) | 181.75 (437.78) | 2.01 (5.02) | 209.77 (472.84) |
| Meat | 20.44 (29.42) | 3317.16 (4598.97) | 20.68 (30.38) | 3556.97 (5071.19) |
| Cereal | 1.23 (2.21) | 400.79 (714.36) | 1.31 (2.29) | 434.01 (725.31) |
| Fruit and veg | 6.30 (6.83) | 3371.04 (3373.43) | 6.54 (6.51) | 3740.15 (3580.1) |
| Tea and coffee | 1.16 (2.68) | 102.88 (245.99) | 1.28 (2.85) | 118.57 (267.78) |
| Juice | 1.03 (2.82) | 797.53 (2109.48) | 0.94 (2.58) | 761.09 (1977.74) |
| Home cooking | 6.98 (11.49) | 2190.78 (3779.55) | 7.24 (11.75) | 2424.04 (4147.5) |
| Biscuits and bakery | 7.68 (7.92) | 2651.38 (2491.34) | 7.80 (8.03) | 2801.3 (2572.72) |
| Crisps and snacks | 1.64 (2.7) | 245.49 (421.11) | 1.54 (2.54) | 235.1 (397.06) |
| Soft drinks | 4.17 (7.17) | 5837.49 (9588.02) | 3.5 (6.16) | 5254.16 (8432.75) |
| Confectionery | 3.09 (4.9) | 842.19 (1419.95) | 2.93 (4.64) | 805.35 (1347.29) |
| Slimming | 0.07 (1.61) | 7.00 (207.28) | 0.08 (1.99) | 6.08 (179.53) |

Table shows mean with standard deviation in parentheses.

**Table A2. Influence of MUP on Overall Household Food Purchases of Category**

|  | Percent | Percent |
| --- | --- | --- |
|  | Spend | Volume |
| Post-MUP | 0.005 [-0.007,0.016] | 0.018^***^ [0.007,0.028] |
| Exposed to MUP in Scotland | -0.010^**^ [-0.019,-0.000] | -0.008 [-0.017,0.002] |
| Age of shopper | 0.050^***^ [0.032,0.068] | 0.040^***^ [0.023,0.057] |
| Age of shopper squared/100 | -0.050^***^ [-0.063,-0.037] | -0.043^***^ [-0.056,-0.031] |
| Total people in household | 0.059^***^ [0.043,0.075] | 0.056^***^ [0.042,0.070] |
| Children dummy | -0.050^**^ [-0.096,-0.004] | -0.029 [-0.076,0.019] |
| Log years in panel | -0.051^***^ [-0.069,-0.032] | -0.053^***^ [-0.071,-0.035] |
| Spend: Non-food | 0.010^***^ [0.010,0.011] | 0.010^***^ [0.009,0.011] |
| Month of purchase: February | 0.023^***^ [0.015,0.030] | 0.014^***^ [0.007,0.020] |
| Month of purchase: March | 0.041^***^ [0.033,0.048] | 0.020^***^ [0.013,0.027] |
| Month of purchase: April | 0.030^***^ [0.022,0.038] | 0.016^***^ [0.009,0.023] |
| Month of purchase: May | 0.024^***^ [0.013,0.035] | 0.011^**^ [0.001,0.020] |
| Month of purchase: June | 0.015^**^ [0.002,0.027] | 0.001 [-0.010,0.012] |
| Month of purchase: July | 0.010 [-0.003,0.022] | 0.004 [-0.007,0.015] |
| Month of purchase: August | 0.019^***^ [0.007,0.032] | 0.007 [-0.004,0.018] |
| Month of purchase: September | 0.015^**^ [0.002,0.028] | -0.008 [-0.020,0.004] |
| Month of purchase: October | 0.023^***^ [0.010,0.035] | -0.009 [-0.021,0.002] |
| Month of purchase: November | 0.044^***^ [0.031,0.057] | -0.001 [-0.013,0.011] |
| Month of purchase: December | 0.221^***^ [0.207,0.235] | 0.108^***^ [0.095,0.120] |
| Observations | 687059 | 687059 |
| Households | 8051 | 8051 |
| Pseudo-R^2^ | 0.407 | 0.487 |

95% confidence intervals in brackets ^*^ *p* < 0.10, ^**^ *p* < 0.05, ^***^ *p* < 0.01

**Table A3. Influence of MUP on Household Food Purchases of Category: Canned Food**

|  | Percent | Percent |
| --- | --- | --- |
|  | Spend | Volume |
| Post-MUP | 0.002 [-0.033,0.037] | -0.026 [-0.059,0.006] |
| Exposed to MUP in Scotland | 0.010 [-0.019,0.040] | 0.005 [-0.021,0.031] |
| Age of shopper | -0.024 [-0.078,0.029] | 0.014 [-0.035,0.063] |
| Age of shopper squared/100 | 0.017 [-0.023,0.056] | 0.002 [-0.031,0.035] |
| Total people in household | 0.073^***^ [0.027,0.119] | 0.082^***^ [0.041,0.122] |
| Children dummy | -0.063 [-0.199,0.074] | -0.063 [-0.170,0.044] |
| Log years in panel | -0.017 [-0.062,0.028] | -0.014 [-0.060,0.031] |
| Spend: Non-food | 0.014^***^ [0.012,0.015] | 0.014^***^ [0.013,0.015] |
| Month of purchase: February | -0.058^***^ [-0.079,-0.036] | -0.067^***^ [-0.086,-0.048] |
| Month of purchase: March | -0.099^***^ [-0.120,-0.077] | -0.120^***^ [-0.139,-0.101] |
| Month of purchase: April | -0.151^***^ [-0.173,-0.130] | -0.176^***^ [-0.195,-0.157] |
| Month of purchase: May | -0.181^***^ [-0.215,-0.146] | -0.228^***^ [-0.260,-0.196] |
| Month of purchase: June | -0.209^***^ [-0.249,-0.169] | -0.275^***^ [-0.312,-0.238] |
| Month of purchase: July | -0.181^***^ [-0.221,-0.142] | -0.251^***^ [-0.288,-0.215] |
| Month of purchase: August | -0.126^***^ [-0.163,-0.089] | -0.169^***^ [-0.204,-0.134] |
| Month of purchase: September | -0.099^***^ [-0.139,-0.060] | -0.109^***^ [-0.145,-0.073] |
| Month of purchase: October | -0.059^***^ [-0.098,-0.021] | -0.060^***^ [-0.095,-0.024] |
| Month of purchase: November | -0.078^***^ [-0.117,-0.039] | -0.060^***^ [-0.095,-0.024] |
| Month of purchase: December | -0.128^***^ [-0.168,-0.088] | -0.143^***^ [-0.180,-0.106] |
| Observations | 685356 | 685356 |
| Households | 8012 | 8012 |
| Pseudo-R^2^ | 0.182 | 0.257 |

95% confidence intervals in brackets ^*^ *p* < 0.10, ^**^ *p* < 0.05, ^***^ *p* < 0.01

**Table A4. Influence of MUP on Household Food Purchases of Category: Convenience Food**

|  | Percent | Percent |
| --- | --- | --- |
|  | Spend | Volume |
| Post-MUP | -0.002 [-0.023,0.019] | 0.001 [-0.019,0.022] |
| Exposed to MUP in Scotland | -0.006 [-0.023,0.012] | 0.002 [-0.015,0.020] |
| Age of shopper | 0.005 [-0.029,0.040] | 0.012 [-0.021,0.045] |
| Age of shopper squared/100 | -0.007 [-0.032,0.019] | -0.004 [-0.028,0.020] |
| Total people in household | 0.069^***^ [0.045,0.093] | 0.061^***^ [0.038,0.083] |
| Children dummy | -0.098^**^ [-0.185,-0.012] | -0.081^*^ [-0.169,0.007] |
| Log years in panel | -0.034^**^ [-0.067,-0.000] | -0.042^***^ [-0.074,-0.010] |
| Spend: Non-food | 0.010^***^ [0.009,0.010] | 0.010^***^ [0.009,0.011] |
| Month of purchase: February | 0.005 [-0.008,0.018] | -0.003 [-0.016,0.009] |
| Month of purchase: March | -0.013^*^ [-0.027,0.001] | -0.016^**^ [-0.028,-0.003] |
| Month of purchase: April | 0.007 [-0.007,0.020] | -0.007 [-0.020,0.006] |
| Month of purchase: May | 0.022^**^ [0.002,0.042] | -0.010 [-0.030,0.009] |
| Month of purchase: June | 0.007 [-0.015,0.030] | -0.022^**^ [-0.045,-0.000] |
| Month of purchase: July | 0.004 [-0.019,0.027] | -0.033^***^ [-0.055,-0.011] |
| Month of purchase: August | -0.004 [-0.026,0.018] | -0.028^***^ [-0.050,-0.007] |
| Month of purchase: September | -0.005 [-0.029,0.018] | -0.022^*^ [-0.045,0.000] |
| Month of purchase: October | 0.003 [-0.020,0.026] | -0.015 [-0.037,0.007] |
| Month of purchase: November | 0.001 [-0.023,0.025] | -0.022^*^ [-0.044,0.001] |
| Month of purchase: December | 0.317^***^ [0.291,0.342] | 0.164^***^ [0.140,0.188] |
| Observations | 686990 | 686990 |
| Households | 8046 | 8046 |
| Pseudo-R^2^ | 0.324 | 0.363 |

95% confidence intervals in brackets ^*^ *p* < 0.10, ^**^ *p* < 0.05, ^***^ *p* < 0.01

**Table A5. Influence of MUP on Household Food Purchases of Category: Rice and Pasta**

|  | Percent | Percent |
| --- | --- | --- |
|  | Spend | Volume |
| Post-MUP | -0.020 [-0.062,0.022] | -0.016 [-0.062,0.029] |
| Exposed to MUP in Scotland | -0.016 [-0.048,0.016] | 0.008 [-0.022,0.037] |
| Age of shopper | 0.068^**^ [0.003,0.132] | 0.087^***^ [0.023,0.150] |
| Age of shopper squared/100 | -0.040^*^ [-0.086,0.007] | -0.042^*^ [-0.086,0.002] |
| Total people in household | 0.080^***^ [0.040,0.120] | 0.066^***^ [0.025,0.107] |
| Children dummy | 0.001 [-0.123,0.126] | 0.079 [-0.059,0.217] |
| Log years in panel | -0.055^**^ [-0.109,-0.001] | -0.050^**^ [-0.097,-0.003] |
| Spend: Non-food | 0.014^***^ [0.013,0.015] | 0.015^***^ [0.013,0.016] |
| Month of purchase: February | -0.090^***^ [-0.114,-0.065] | -0.065^***^ [-0.091,-0.039] |
| Month of purchase: March | -0.117^***^ [-0.143,-0.090] | -0.082^***^ [-0.111,-0.054] |
| Month of purchase: April | -0.099^***^ [-0.125,-0.072] | -0.082^***^ [-0.109,-0.054] |
| Month of purchase: May | -0.135^***^ [-0.174,-0.097] | -0.110^***^ [-0.153,-0.066] |
| Month of purchase: June | -0.146^***^ [-0.192,-0.101] | -0.137^***^ [-0.188,-0.086] |
| Month of purchase: July | -0.129^***^ [-0.175,-0.083] | -0.130^***^ [-0.182,-0.079] |
| Month of purchase: August | -0.066^***^ [-0.110,-0.022] | -0.059^**^ [-0.108,-0.009] |
| Month of purchase: September | -0.081^***^ [-0.126,-0.036] | -0.045^*^ [-0.095,0.006] |
| Month of purchase: October | -0.092^***^ [-0.137,-0.046] | -0.056^**^ [-0.106,-0.005] |
| Month of purchase: November | -0.111^***^ [-0.158,-0.063] | -0.075^***^ [-0.126,-0.024] |
| Month of purchase: December | -0.249^***^ [-0.297,-0.201] | -0.209^***^ [-0.262,-0.156] |
| Observations | 673182 | 673182 |
| Households | 7878 | 7878 |
| Pseudo-R^2^ | 0.193 | 0.255 |

95% confidence intervals in brackets ^*^ *p* < 0.10, ^**^ *p* < 0.05, ^***^ *p* < 0.01

**Table A6. Influence of MUP on Household Food Purchases of Category: Dairy**

|  | Percent | Percent |
| --- | --- | --- |
|  | Spend | Volume |
| Post-MUP | 0.015^**^ [0.000,0.029] | -0.001 [-0.015,0.013] |
| Exposed to MUP in Scotland | -0.014^**^ [-0.027,-0.002] | -0.013^*^ [-0.028,0.001] |
| Age of shopper | 0.014 [-0.010,0.037] | 0.024^*^ [-0.002,0.050] |
| Age of shopper squared/100 | -0.023^***^ [-0.041,-0.006] | -0.032^***^ [-0.051,-0.013] |
| Total people in household | 0.056^***^ [0.035,0.076] | 0.057^***^ [0.032,0.081] |
| Children dummy | 0.012 [-0.047,0.071] | 0.021 [-0.043,0.085] |
| Log years in panel | -0.030^**^ [-0.053,-0.007] | -0.023^*^ [-0.051,0.004] |
| Spend: Non-food | 0.009^***^ [0.008,0.010] | 0.007^***^ [0.007,0.008] |
| Month of purchase: February | 0.027^***^ [0.019,0.035] | 0.010^**^ [0.002,0.018] |
| Month of purchase: March | 0.023^***^ [0.015,0.032] | -0.002 [-0.010,0.007] |
| Month of purchase: April | 0.011^**^ [0.001,0.020] | -0.008^*^ [-0.017,0.001] |
| Month of purchase: May | -0.002 [-0.015,0.011] | -0.017^***^ [-0.030,-0.004] |
| Month of purchase: June | -0.013^*^ [-0.029,0.002] | -0.031^***^ [-0.045,-0.016] |
| Month of purchase: July | -0.014^*^ [-0.030,0.002] | -0.040^***^ [-0.055,-0.025] |
| Month of purchase: August | -0.000 [-0.015,0.015] | -0.023^***^ [-0.038,-0.009] |
| Month of purchase: September | 0.004 [-0.011,0.020] | -0.025^***^ [-0.040,-0.010] |
| Month of purchase: October | -0.004 [-0.020,0.011] | -0.036^***^ [-0.051,-0.021] |
| Month of purchase: November | 0.008 [-0.007,0.024] | -0.026^***^ [-0.041,-0.011] |
| Month of purchase: December | 0.138^***^ [0.121,0.155] | -0.003 [-0.019,0.013] |
| Observations | 686878 | 686878 |
| Households | 8045 | 8045 |
| Pseudo-R^2^ | 0.271 | 0.495 |

95% confidence intervals in brackets ^*^ *p* < 0.10, ^**^ *p* < 0.05, ^***^ *p* < 0.01

**Table A7. Influence of MUP on Household Food Purchases of Category: Fish**

|  | Percent | Percent |
| --- | --- | --- |
|  | Spend | Volume |
| Post-MUP | -0.024 [-0.071,0.023] | -0.005 [-0.047,0.037] |
| Exposed to MUP in Scotland | -0.014 [-0.051,0.024] | -0.041^**^ [-0.075,-0.006] |
| Age of shopper | 0.117^***^ [0.046,0.189] | 0.125^***^ [0.061,0.190] |
| Age of shopper squared/100 | -0.051^**^ [-0.102,-0.000] | -0.052^**^ [-0.096,-0.007] |
| Total people in household | 0.070^**^ [0.014,0.127] | 0.080^***^ [0.033,0.128] |
| Children dummy | -0.046 [-0.218,0.126] | -0.052 [-0.207,0.104] |
| Log years in panel | -0.052 [-0.124,0.020] | -0.056^*^ [-0.118,0.005] |
| Spend: Non-food | 0.010^***^ [0.009,0.011] | 0.010^***^ [0.010,0.011] |
| Month of purchase: February | 0.010 [-0.018,0.039] | -0.010 [-0.035,0.016] |
| Month of purchase: March | 0.064^***^ [0.036,0.092] | 0.050^***^ [0.024,0.076] |
| Month of purchase: April | 0.068^***^ [0.038,0.099] | 0.051^***^ [0.024,0.078] |
| Month of purchase: May | 0.080^***^ [0.038,0.123] | 0.047^**^ [0.008,0.085] |
| Month of purchase: June | 0.088^***^ [0.037,0.139] | 0.031 [-0.015,0.076] |
| Month of purchase: July | 0.074^***^ [0.024,0.125] | 0.037 [-0.008,0.083] |
| Month of purchase: August | 0.085^***^ [0.037,0.134] | 0.061^***^ [0.017,0.105] |
| Month of purchase: September | 0.043 [-0.009,0.095] | 0.033 [-0.014,0.080] |
| Month of purchase: October | 0.018 [-0.034,0.069] | -0.001 [-0.047,0.044] |
| Month of purchase: November | -0.006 [-0.058,0.046] | -0.024 [-0.071,0.024] |
| Month of purchase: December | 0.306^***^ [0.251,0.361] | 0.120^***^ [0.069,0.170] |
| Observations | 651369 | 651369 |
| Households | 7525 | 7525 |
| Pseudo-R^2^ | 0.279 | 0.262 |

95% confidence intervals in brackets ^*^ *p* < 0.10, ^**^ *p* < 0.05, ^***^ *p* < 0.01

**Table A8. Influence of MUP on Household Food Purchases of Category: Meat**

|  | Percent | Percent |
| --- | --- | --- |
|  | Spend | Volume |
| Post-MUP | -0.013 [-0.037,0.012] | 0.007 [-0.017,0.030] |
| Exposed to MUP in Scotland | -0.012 [-0.033,0.008] | -0.002 [-0.021,0.018] |
| Age of shopper | 0.109^***^ [0.070,0.148] | 0.116^***^ [0.078,0.154] |
| Age of shopper squared/100 | -0.102^***^ [-0.130,-0.074] | -0.099^***^ [-0.126,-0.073] |
| Total people in household | 0.064^***^ [0.035,0.094] | 0.058^***^ [0.029,0.086] |
| Children dummy | -0.093^**^ [-0.180,-0.006] | -0.064 [-0.151,0.023] |
| Log years in panel | -0.068^***^ [-0.107,-0.029] | -0.069^***^ [-0.105,-0.032] |
| Spend: Non-food | 0.011^***^ [0.010,0.012] | 0.011^***^ [0.010,0.012] |
| Month of purchase: February | 0.013^*^ [-0.002,0.028] | 0.007 [-0.008,0.021] |
| Month of purchase: March | 0.051^***^ [0.036,0.067] | 0.036^***^ [0.022,0.051] |
| Month of purchase: April | 0.020^**^ [0.004,0.036] | 0.013^*^ [-0.002,0.029] |
| Month of purchase: May | 0.004 [-0.020,0.028] | -0.014 [-0.036,0.009] |
| Month of purchase: June | -0.004 [-0.031,0.023] | -0.027^**^ [-0.053,-0.000] |
| Month of purchase: July | -0.023 [-0.050,0.005] | -0.034^**^ [-0.061,-0.008] |
| Month of purchase: August | 0.007 [-0.020,0.034] | -0.010 [-0.036,0.017] |
| Month of purchase: September | 0.008 [-0.020,0.036] | -0.006 [-0.033,0.021] |
| Month of purchase: October | 0.010 [-0.018,0.038] | -0.013 [-0.039,0.013] |
| Month of purchase: November | 0.044^***^ [0.015,0.072] | 0.029^**^ [0.001,0.056] |
| Month of purchase: December | 0.224^***^ [0.193,0.255] | 0.168^***^ [0.138,0.197] |
| Observations | 673081 | 673081 |
| Households | 7864 | 7864 |
| Pseudo-R^2^ | 0.317 | 0.353 |

95% confidence intervals in brackets ^*^ *p* < 0.10, ^**^ *p* < 0.05, ^***^ *p* < 0.01

**Table A9. Influence of MUP on Household Food Purchases of Category: Cereal**

|  | Percent | Percent |
| --- | --- | --- |
|  | Spend | Volume |
| Post-MUP | 0.005 [-0.027,0.037] | 0.017 [-0.012,0.047] |
| Exposed to MUP in Scotland | -0.035^***^ [-0.060,-0.010] | -0.020^*^ [-0.044,0.003] |
| Age of shopper | 0.019 [-0.029,0.067] | 0.039^*^ [-0.005,0.083] |
| Age of shopper squared/100 | -0.048^***^ [-0.080,-0.016] | -0.060^***^ [-0.089,-0.031] |
| Total people in household | 0.028 [-0.006,0.061] | 0.026 [-0.008,0.060] |
| Children dummy | 0.024 [-0.081,0.129] | 0.067 [-0.032,0.166] |
| Log years in panel | -0.026 [-0.070,0.017] | -0.039^*^ [-0.078,0.000] |
| Spend: Non-food | 0.013^***^ [0.012,0.015] | 0.013^***^ [0.012,0.015] |
| Month of purchase: February | 0.024^**^ [0.004,0.044] | 0.016 [-0.003,0.036] |
| Month of purchase: March | 0.010 [-0.009,0.030] | 0.000 [-0.018,0.019] |
| Month of purchase: April | -0.008 [-0.028,0.013] | -0.015 [-0.034,0.005] |
| Month of purchase: May | 0.014 [-0.017,0.046] | -0.019 [-0.048,0.010] |
| Month of purchase: June | -0.005 [-0.041,0.031] | -0.034^**^ [-0.067,-0.001] |
| Month of purchase: July | -0.007 [-0.043,0.029] | -0.043^***^ [-0.075,-0.010] |
| Month of purchase: August | 0.009 [-0.025,0.044] | -0.019 [-0.051,0.013] |
| Month of purchase: September | 0.022 [-0.015,0.058] | -0.006 [-0.039,0.028] |
| Month of purchase: October | 0.002 [-0.034,0.037] | -0.022 [-0.055,0.011] |
| Month of purchase: November | -0.031^*^ [-0.067,0.005] | -0.058^***^ [-0.091,-0.025] |
| Month of purchase: December | -0.100^***^ [-0.138,-0.062] | -0.150^***^ [-0.185,-0.116] |
| Observations | 681337 | 681337 |
| Households | 7949 | 7949 |
| Pseudo-R^2^ | 0.196 | 0.286 |

95% confidence intervals in brackets ^*^ *p* < 0.10, ^**^ *p* < 0.05, ^***^ *p* < 0.01

**Table A10. Influence of MUP on Household Food Purchases of Category: Fruit and Vegetables**

|  | Percent | Percent |
| --- | --- | --- |
|  | Spend | Volume |
| Post-MUP | 0.058^***^ [0.041,0.075] | 0.006 [-0.010,0.022] |
| Exposed to MUP in Scotland | -0.025^***^ [-0.043,-0.008] | -0.012 [-0.028,0.003] |
| Age of shopper | 0.019 [-0.010,0.049] | 0.047^***^ [0.020,0.073] |
| Age of shopper squared/100 | -0.058^***^ [-0.081,-0.035] | -0.049^***^ [-0.069,-0.030] |
| Total people in household | 0.031^*^ [-0.001,0.064] | 0.041^***^ [0.015,0.067] |
| Children dummy | 0.012 [-0.083,0.108] | 0.001 [-0.081,0.084] |
| Log years in panel | -0.046^***^ [-0.075,-0.017] | -0.048^***^ [-0.076,-0.020] |
| Spend: Non-food | 0.008^***^ [0.007,0.008] | 0.008^***^ [0.008,0.008] |
| Month of purchase: February | 0.000 [-0.010,0.010] | -0.012^**^ [-0.021,-0.002] |
| Month of purchase: March | 0.005 [-0.005,0.016] | 0.007 [-0.003,0.017] |
| Month of purchase: April | 0.036^***^ [0.024,0.047] | -0.006 [-0.016,0.004] |
| Month of purchase: May | 0.071^***^ [0.056,0.087] | -0.000 [-0.014,0.014] |
| Month of purchase: June | 0.079^***^ [0.061,0.097] | 0.011 [-0.006,0.028] |
| Month of purchase: July | 0.053^***^ [0.035,0.071] | 0.007 [-0.010,0.025] |
| Month of purchase: August | 0.006 [-0.012,0.024] | -0.012 [-0.029,0.005] |
| Month of purchase: September | -0.058^***^ [-0.077,-0.039] | -0.052^***^ [-0.070,-0.035] |
| Month of purchase: October | -0.090^***^ [-0.108,-0.072] | -0.062^***^ [-0.079,-0.045] |
| Month of purchase: November | -0.117^***^ [-0.135,-0.098] | -0.077^***^ [-0.094,-0.060] |
| Month of purchase: December | -0.150^***^ [-0.169,-0.130] | -0.010 [-0.029,0.008] |
| Observations | 686271 | 686271 |
| Households | 8037 | 8037 |
| Pseudo-R^2^ | 0.337 | 0.441 |

95% confidence intervals in brackets ^*^ *p* < 0.10, ^**^ *p* < 0.05, ^***^ *p* < 0.01

**Table A11. Influence of MUP on Household Food Purchases of Category: Tea and Coffee**

|  | Percent | Percent |
| --- | --- | --- |
|  | Spend | Volume |
| Post-MUP | -0.054^**^ [-0.096,-0.012] | -0.054^***^ [-0.095,-0.014] |
| Exposed to MUP in Scotland | 0.007 [-0.026,0.039] | 0.022 [-0.006,0.050] |
| Age of shopper | 0.077^**^ [0.015,0.139] | 0.124^***^ [0.066,0.181] |
| Age of shopper squared/100 | -0.038^*^ [-0.080,0.004] | -0.055^***^ [-0.093,-0.017] |
| Total people in household | 0.082^***^ [0.038,0.127] | 0.054^**^ [0.010,0.098] |
| Children dummy | 0.094 [-0.055,0.244] | 0.047 [-0.082,0.177] |
| Log years in panel | -0.057^**^ [-0.109,-0.004] | -0.044^*^ [-0.093,0.005] |
| Spend: Non-food | 0.015^***^ [0.013,0.016] | 0.015^***^ [0.014,0.017] |
| Month of purchase: February | -0.009 [-0.036,0.018] | -0.012 [-0.040,0.016] |
| Month of purchase: March | -0.004 [-0.031,0.022] | -0.015 [-0.042,0.012] |
| Month of purchase: April | -0.026^**^ [-0.051,-0.000] | -0.039^***^ [-0.065,-0.012] |
| Month of purchase: May | -0.048^**^ [-0.089,-0.006] | -0.078^***^ [-0.120,-0.036] |
| Month of purchase: June | -0.098^***^ [-0.144,-0.051] | -0.094^***^ [-0.142,-0.047] |
| Month of purchase: July | -0.106^***^ [-0.153,-0.059] | -0.123^***^ [-0.170,-0.075] |
| Month of purchase: August | -0.042^*^ [-0.087,0.004] | -0.078^***^ [-0.123,-0.033] |
| Month of purchase: September | 0.012 [-0.036,0.061] | 0.014 [-0.033,0.061] |
| Month of purchase: October | -0.003 [-0.049,0.043] | 0.017 [-0.028,0.062] |
| Month of purchase: November | 0.070^***^ [0.024,0.117] | 0.084^***^ [0.037,0.132] |
| Month of purchase: December | 0.073^***^ [0.025,0.121] | 0.049^**^ [0.001,0.097] |
| Observations | 672241 | 672241 |
| Households | 7772 | 7772 |
| Pseudo-R^2^ | 0.195 | 0.207 |

95% confidence intervals in brackets ^*^ *p* < 0.10, ^**^ *p* < 0.05, ^***^ *p* < 0.01

**Table A12. Influence of MUP on Household Food Purchases of Category: Juice**

|  | Percent | Percent |
| --- | --- | --- |
|  | Spend | Volume |
| Post-MUP | 0.026 [-0.023,0.076] | 0.048^**^ [0.000,0.095] |
| Exposed to MUP in Scotland | -0.012 [-0.062,0.038] | -0.024 [-0.074,0.026] |
| Age of shopper | -0.015 [-0.101,0.070] | -0.022 [-0.106,0.062] |
| Age of shopper squared/100 | -0.039 [-0.107,0.030] | -0.038 [-0.105,0.029] |
| Total people in household | 0.016 [-0.070,0.102] | 0.022 [-0.067,0.111] |
| Children dummy | -0.042 [-0.220,0.135] | -0.068 [-0.252,0.115] |
| Log years in panel | -0.028 [-0.110,0.054] | -0.018 [-0.106,0.069] |
| Spend: Non-food | 0.010^***^ [0.009,0.011] | 0.010^***^ [0.009,0.011] |
| Month of purchase: February | 0.009 [-0.021,0.038] | 0.010 [-0.017,0.037] |
| Month of purchase: March | 0.044^***^ [0.013,0.076] | 0.025^*^ [-0.004,0.054] |
| Month of purchase: April | 0.034^**^ [0.003,0.065] | 0.040^***^ [0.011,0.069] |
| Month of purchase: May | 0.039^*^ [-0.001,0.080] | 0.036^*^ [-0.004,0.075] |
| Month of purchase: June | 0.021 [-0.027,0.068] | 0.008 [-0.037,0.053] |
| Month of purchase: July | 0.010 [-0.039,0.059] | -0.010 [-0.056,0.036] |
| Month of purchase: August | 0.008 [-0.039,0.056] | -0.005 [-0.052,0.043] |
| Month of purchase: September | -0.041 [-0.091,0.010] | -0.063^**^ [-0.111,-0.015] |
| Month of purchase: October | -0.082^***^ [-0.132,-0.032] | -0.099^***^ [-0.147,-0.050] |
| Month of purchase: November | -0.085^***^ [-0.136,-0.033] | -0.109^***^ [-0.158,-0.059] |
| Month of purchase: December | 0.011 [-0.040,0.062] | 0.001 [-0.047,0.050] |
| Observations | 532639 | 532639 |
| Households | 6159 | 6159 |
| Pseudo-R^2^ | 0.354 | 0.430 |

95% confidence intervals in brackets ^*^ *p* < 0.10, ^**^ *p* < 0.05, ^***^ *p* < 0.01

**Table A13. Influence of MUP on Household Food Purchases of Category: Home Cooking and Condiments**

|  | Percent | Percent |
| --- | --- | --- |
|  | Spend | Volume |
| Post-MUP | -0.031^*^ [-0.063,0.001] | -0.020 [-0.052,0.013] |
| Exposed to MUP in Scotland | -0.017 [-0.040,0.006] | -0.008 [-0.031,0.016] |
| Age of shopper | 0.045^*^ [-0.003,0.094] | 0.065^***^ [0.016,0.114] |
| Age of shopper squared/100 | -0.046^***^ [-0.079,-0.013] | -0.056^***^ [-0.090,-0.023] |
| Total people in household | 0.052^***^ [0.022,0.083] | 0.053^***^ [0.023,0.084] |
| Children dummy | 0.003 [-0.107,0.113] | -0.020 [-0.129,0.089] |
| Log years in panel | -0.010 [-0.048,0.029] | -0.024 [-0.064,0.017] |
| Spend: Non-food | 0.014^***^ [0.013,0.015] | 0.014^***^ [0.013,0.015] |
| Month of purchase: February | 0.003 [-0.016,0.022] | 0.010 [-0.010,0.030] |
| Month of purchase: March | -0.063^***^ [-0.083,-0.043] | -0.073^***^ [-0.094,-0.053] |
| Month of purchase: April | -0.048^***^ [-0.067,-0.029] | -0.045^***^ [-0.065,-0.025] |
| Month of purchase: May | -0.070^***^ [-0.100,-0.039] | -0.066^***^ [-0.098,-0.034] |
| Month of purchase: June | -0.103^***^ [-0.139,-0.067] | -0.083^***^ [-0.119,-0.046] |
| Month of purchase: July | -0.100^***^ [-0.137,-0.064] | -0.098^***^ [-0.134,-0.061] |
| Month of purchase: August | -0.032^*^ [-0.067,0.003] | -0.021 [-0.057,0.014] |
| Month of purchase: September | -0.021 [-0.057,0.015] | -0.001 [-0.038,0.036] |
| Month of purchase: October | 0.003 [-0.032,0.038] | 0.003 [-0.033,0.038] |
| Month of purchase: November | 0.004 [-0.032,0.040] | -0.020 [-0.056,0.016] |
| Month of purchase: December | 0.104^***^ [0.066,0.141] | 0.081^***^ [0.043,0.119] |
| Observations | 686726 | 686726 |
| Households | 8043 | 8043 |
| Pseudo-R^2^ | 0.225 | 0.277 |

95% confidence intervals in brackets ^*^ *p* < 0.10, ^**^ *p* < 0.05, ^***^ *p* < 0.01

**Table A14. Influence of MUP on Household Food Purchases of Category: Biscuits and Bakery**

|  | Percent | Percent |
| --- | --- | --- |
|  | Spend | Volume |
| Post-MUP | 0.007 [-0.010,0.024] | 0.001 [-0.014,0.016] |
| Exposed to MUP in Scotland | -0.005 [-0.020,0.011] | 0.001 [-0.013,0.015] |
| Age of shopper | 0.071^***^ [0.043,0.099] | 0.050^***^ [0.024,0.077] |
| Age of shopper squared/100 | -0.058^***^ [-0.078,-0.037] | -0.037^***^ [-0.057,-0.017] |
| Total people in household | 0.063^***^ [0.041,0.084] | 0.067^***^ [0.047,0.087] |
| Children dummy | -0.049 [-0.108,0.011] | -0.061^**^ [-0.114,-0.009] |
| Log years in panel | -0.047^***^ [-0.074,-0.021] | -0.049^***^ [-0.073,-0.025] |
| Spend: Non-food | 0.009^***^ [0.008,0.010] | 0.008^***^ [0.007,0.009] |
| Month of purchase: February | 0.067^***^ [0.056,0.077] | 0.051^***^ [0.042,0.061] |
| Month of purchase: March | 0.119^***^ [0.107,0.130] | 0.093^***^ [0.083,0.103] |
| Month of purchase: April | 0.081^***^ [0.069,0.093] | 0.062^***^ [0.052,0.072] |
| Month of purchase: May | 0.077^***^ [0.061,0.093] | 0.050^***^ [0.036,0.064] |
| Month of purchase: June | 0.058^***^ [0.040,0.076] | 0.025^***^ [0.009,0.042] |
| Month of purchase: July | 0.051^***^ [0.033,0.070] | 0.021^**^ [0.004,0.038] |
| Month of purchase: August | 0.073^***^ [0.055,0.091] | 0.042^***^ [0.025,0.058] |
| Month of purchase: September | 0.074^***^ [0.056,0.093] | 0.032^***^ [0.015,0.049] |
| Month of purchase: October | 0.102^***^ [0.083,0.120] | 0.052^***^ [0.036,0.068] |
| Month of purchase: November | 0.171^***^ [0.152,0.191] | 0.090^***^ [0.073,0.107] |
| Month of purchase: December | 0.339^***^ [0.318,0.359] | 0.188^***^ [0.170,0.205] |
| Observations | 686968 | 686968 |
| Households | 8046 | 8046 |
| Pseudo-R^2^ | 0.288 | 0.428 |

95% confidence intervals in brackets ^*^ *p* < 0.10, ^**^ *p* < 0.05, ^***^ *p* < 0.01

**Table A15. Influence of MUP on Household Food Purchases of Category: Crisps and Snacks**

|  | Percent | Percent |
| --- | --- | --- |
|  | Spend | Volume |
| Post-MUP | 0.026^*^ [-0.003,0.054] | 0.002 [-0.027,0.031] |
| Exposed to MUP in Scotland | 0.025^**^ [0.002,0.049] | 0.020^*^ [-0.003,0.044] |
| Age of shopper | 0.137^***^ [0.091,0.184] | 0.150^***^ [0.102,0.197] |
| Age of shopper squared/100 | -0.126^***^ [-0.163,-0.089] | -0.124^***^ [-0.161,-0.087] |
| Total people in household | 0.047^***^ [0.013,0.080] | 0.055^***^ [0.019,0.090] |
| Children dummy | -0.096^*^ [-0.207,0.015] | -0.110^**^ [-0.217,-0.002] |
| Log years in panel | -0.089^***^ [-0.127,-0.050] | -0.086^***^ [-0.125,-0.046] |
| Spend: Non-food | 0.011^***^ [0.009,0.012] | 0.011^***^ [0.010,0.012] |
| Month of purchase: February | 0.093^***^ [0.075,0.111] | 0.091^***^ [0.072,0.110] |
| Month of purchase: March | 0.130^***^ [0.110,0.149] | 0.121^***^ [0.101,0.141] |
| Month of purchase: April | 0.121^***^ [0.101,0.141] | 0.118^***^ [0.098,0.139] |
| Month of purchase: May | 0.121^***^ [0.093,0.150] | 0.131^***^ [0.102,0.160] |
| Month of purchase: June | 0.120^***^ [0.088,0.152] | 0.133^***^ [0.100,0.165] |
| Month of purchase: July | 0.119^***^ [0.086,0.152] | 0.135^***^ [0.102,0.168] |
| Month of purchase: August | 0.139^***^ [0.107,0.171] | 0.158^***^ [0.125,0.190] |
| Month of purchase: September | 0.129^***^ [0.096,0.162] | 0.132^***^ [0.098,0.165] |
| Month of purchase: October | 0.164^***^ [0.131,0.197] | 0.188^***^ [0.154,0.222] |
| Month of purchase: November | 0.211^***^ [0.177,0.245] | 0.234^***^ [0.200,0.268] |
| Month of purchase: December | 0.486^***^ [0.451,0.522] | 0.535^***^ [0.499,0.571] |
| Observations | 677342 | 677342 |
| Households | 7912 | 7912 |
| Pseudo-R^2^ | 0.233 | 0.332 |

95% confidence intervals in brackets ^*^ *p* < 0.10, ^**^ *p* < 0.05, ^***^ *p* < 0.01

**Table A16. Influence of MUP on Household Food Purchases of Category: Soft Drinks**

|  | Percent | Percent |
| --- | --- | --- |
|  | Spend | Volume |
| Post-MUP | 0.108^***^ [0.078,0.138] | 0.094^***^ [0.065,0.123] |
| Exposed to MUP in Scotland | -0.018 [-0.047,0.011] | -0.025^*^ [-0.051,0.002] |
| Age of shopper | 0.011 [-0.043,0.065] | 0.026 [-0.022,0.074] |
| Age of shopper squared/100 | -0.038^*^ [-0.083,0.007] | -0.054^***^ [-0.090,-0.017] |
| Total people in household | 0.065^***^ [0.031,0.100] | 0.055^***^ [0.021,0.089] |
| Children dummy | -0.063 [-0.167,0.042] | -0.040 [-0.162,0.082] |
| Log years in panel | -0.130^***^ [-0.172,-0.087] | -0.113^***^ [-0.154,-0.073] |
| Spend: Non-food | 0.011^***^ [0.010,0.012] | 0.011^***^ [0.009,0.012] |
| Month of purchase: February | 0.036^***^ [0.016,0.056] | 0.030^***^ [0.013,0.047] |
| Month of purchase: March | 0.082^***^ [0.061,0.104] | 0.060^***^ [0.041,0.078] |
| Month of purchase: April | 0.120^***^ [0.098,0.142] | 0.097^***^ [0.078,0.116] |
| Month of purchase: May | 0.130^***^ [0.102,0.159] | 0.121^***^ [0.095,0.147] |
| Month of purchase: June | 0.137^***^ [0.105,0.169] | 0.123^***^ [0.095,0.152] |
| Month of purchase: July | 0.199^***^ [0.167,0.232] | 0.169^***^ [0.139,0.199] |
| Month of purchase: August | 0.133^***^ [0.101,0.164] | 0.102^***^ [0.071,0.132] |
| Month of purchase: September | 0.052^***^ [0.017,0.086] | 0.027 [-0.005,0.059] |
| Month of purchase: October | 0.038^**^ [0.004,0.072] | 0.014 [-0.017,0.046] |
| Month of purchase: November | 0.068^***^ [0.035,0.102] | 0.029^*^ [-0.002,0.061] |
| Month of purchase: December | 0.359^***^ [0.323,0.395] | 0.271^***^ [0.238,0.304] |
| Observations | 682226 | 682226 |
| Households | 7977 | 7977 |
| Pseudo-R^2^ | 0.329 | 0.449 |

95% confidence intervals in brackets ^*^ *p* < 0.10, ^**^ *p* < 0.05, ^***^ *p* < 0.01

**Table A17. Influence of MUP on Household Food Purchases of Category: Confectionery**

|  | Percent | Percent |
| --- | --- | --- |
|  | Spend | Volume |
| Post-MUP | -0.011 [-0.036,0.013] | 0.081^***^ [0.051,0.111] |
| Exposed to MUP in Scotland | 0.005 [-0.017,0.026] | 0.014 [-0.013,0.042] |
| Age of shopper | 0.141^***^ [0.101,0.181] | 0.073^***^ [0.021,0.124] |
| Age of shopper squared/100 | -0.088^***^ [-0.118,-0.058] | -0.093^***^ [-0.131,-0.055] |
| Total people in household | 0.036^**^ [0.004,0.068] | 0.044^***^ [0.011,0.078] |
| Children dummy | 0.016 [-0.079,0.112] | -0.014 [-0.110,0.081] |
| Log years in panel | -0.103^***^ [-0.139,-0.067] | -0.107^***^ [-0.152,-0.061] |
| Spend: Non-food | 0.011^***^ [0.010,0.012] | 0.011^***^ [0.009,0.012] |
| Month of purchase: February | 0.308^***^ [0.289,0.328] | 0.261^***^ [0.238,0.283] |
| Month of purchase: March | 0.600^***^ [0.578,0.622] | 0.410^***^ [0.386,0.435] |
| Month of purchase: April | 0.449^***^ [0.427,0.471] | 0.306^***^ [0.282,0.330] |
| Month of purchase: May | 0.315^***^ [0.290,0.340] | 0.278^***^ [0.247,0.310] |
| Month of purchase: June | 0.374^***^ [0.346,0.403] | 0.308^***^ [0.273,0.343] |
| Month of purchase: July | 0.367^***^ [0.337,0.396] | 0.308^***^ [0.271,0.344] |
| Month of purchase: August | 0.325^***^ [0.296,0.354] | 0.275^***^ [0.240,0.310] |
| Month of purchase: September | 0.410^***^ [0.380,0.441] | 0.294^***^ [0.257,0.331] |
| Month of purchase: October | 0.545^***^ [0.515,0.575] | 0.330^***^ [0.294,0.366] |
| Month of purchase: November | 0.687^***^ [0.654,0.719] | 0.332^***^ [0.295,0.369] |
| Month of purchase: December | 0.848^***^ [0.813,0.882] | 0.382^***^ [0.343,0.421] |
| Observations | 685616 | 685616 |
| Households | 8021 | 8021 |
| Pseudo-R^2^ | 0.251 | 0.358 |

95% confidence intervals in brackets ^*^ *p* < 0.10, ^**^ *p* < 0.05, ^***^ *p* < 0.01

**Table A18. Influence of MUP on** **Household Food Purchases of Category: Slimming**

|  | Percent | Percent |
| --- | --- | --- |
|  | Spend | Volume |
| Post-MUP | 0.470^**^ [0.008,0.932] | 0.780^**^ [0.166,1.394] |
| Exposed to MUP in Scotland | -0.180 [-0.750,0.390] | -0.460 [-1.011,0.091] |
| Age of shopper | 0.147 [-0.590,0.885] | -0.003 [-0.801,0.794] |
| Age of shopper squared/100 | -0.269 [-0.772,0.234] | -0.329 [-0.831,0.173] |
| Total people in household | -0.106 [-1.005,0.793] | -0.018 [-1.252,1.216] |
| Children dummy | 0.372 [-1.228,1.972] | 0.666 [-1.244,2.577] |
| Log years in panel | -0.415 [-0.978,0.148] | -0.161 [-0.837,0.514] |
| Spend: Non-food | 0.016^***^ [0.011,0.021] | 0.015^***^ [0.009,0.020] |
| Month of purchase: February | -0.164 [-0.425,0.097] | -0.271^*^ [-0.550,0.008] |
| Month of purchase: March | -0.266^**^ [-0.531,-0.001] | -0.388^**^ [-0.689,-0.087] |
| Month of purchase: April | -0.101 [-0.391,0.189] | -0.395^***^ [-0.673,-0.117] |
| Month of purchase: May | -0.180 [-0.535,0.174] | -0.360^*^ [-0.765,0.044] |
| Month of purchase: June | -0.227 [-0.601,0.147] | -0.431^**^ [-0.824,-0.039] |
| Month of purchase: July | -0.208 [-0.613,0.197] | -0.308 [-0.767,0.151] |
| Month of purchase: August | -0.081 [-0.470,0.308] | -0.361^*^ [-0.762,0.041] |
| Month of purchase: September | -0.208 [-0.688,0.272] | -0.403 [-0.918,0.112] |
| Month of purchase: October | -0.097 [-0.579,0.385] | -0.240 [-0.791,0.312] |
| Month of purchase: November | -0.176 [-0.651,0.299] | -0.398 [-0.876,0.079] |
| Month of purchase: December | -0.520 [-1.152,0.112] | -0.638 [-1.459,0.183] |
| Observations | 29901 | 29901 |
| Households | 334 | 334 |
| Pseudo-R^2^ | 0.252 | 0.341 |

95% confidence intervals in brackets ^*^ *p* < 0.10, ^**^ *p* < 0.05, ^***^ *p* < 0.01

**Table A19. Test of Common Trends Assumption for Linear and Multiplicative Overall Models**

|  | Spend | Spend | Volume | Volume |
| --- | --- | --- | --- | --- |
|  | Linear | Multiplicative | Linear | Multiplicative |
| Month of purchase:5/17 | -3.113^***^ (0.825) | -0.039^***^ (0.010) | -682.882^**^ (280.061) | -0.022^***^ (0.008) |
| Month of purchase:6/17 | -3.187^***^ (0.824) | -0.040^***^ (0.010) | -697.495^**^ (277.567) | -0.023^***^ (0.008) |
| Month of purchase:7/17 | -3.803^***^ (0.820) | -0.047^***^ (0.010) | -914.634^***^ (281.808) | -0.029^***^ (0.008) |
| Month of purchase:8/17 | -3.481^***^ (0.810) | -0.043^***^ (0.009) | -760.513^***^ (280.441) | -0.024^***^ (0.008) |
| Month of purchase:9/17 | -2.548^***^ (0.848) | -0.032^***^ (0.010) | -893.554^***^ (289.993) | -0.028^***^ (0.009) |
| Month of purchase:10/17 | -2.444^***^ (0.839) | -0.032^***^ (0.010) | -1131.694^***^ (286.633) | -0.037^***^ (0.009) |
| Month of purchase:11/17 | 0.090 (0.850) | -0.001 (0.010) | -585.533^**^ (288.734) | -0.019^**^ (0.009) |
| Month of purchase:12/17 | 15.741^***^ (0.933) | 0.166^***^ (0.010) | 2628.872^***^ (307.367) | 0.075^***^ (0.009) |
| Month of purchase:1/18 | -0.566 (1.029) | -0.004 (0.012) | 247.859 (366.789) | 0.011 (0.011) |
| Month of purchase:2/18 | 1.870^*^ (1.043) | 0.025^**^ (0.012) | 974.360^***^ (368.551) | 0.033^***^ (0.011) |
| Month of purchase:3/18 | 4.565^***^ (1.051) | 0.055^***^ (0.012) | 1400.803^***^ (371.737) | 0.045^***^ (0.011) |
| Month of purchase:4/18 | 0.442 (1.045) | 0.007 (0.012) | 634.516^*^ (371.211) | 0.022^*^ (0.011) |
| Month of purchase:5/18 | 2.066^**^ (1.050) | 0.025^**^ (0.012) | 1201.839^***^ (373.713) | 0.039^***^ (0.011) |
| Month of purchase:6/18 | 1.139 (1.065) | 0.013 (0.013) | 980.851^***^ (379.680) | 0.030^***^ (0.012) |
| Month of purchase:7/18 | -0.144 (1.074) | -0.001 (0.013) | 804.901^**^ (384.585) | 0.027^**^ (0.012) |
| Month of purchase:8/18 | 0.217 (1.063) | 0.005 (0.013) | 633.025^*^ (378.546) | 0.023^*^ (0.012) |
| Month of purchase:9/18 | 1.163 (1.090) | 0.016 (0.013) | 622.992 (384.187) | 0.022^*^ (0.012) |
| Month of purchase:10/18 | 1.598 (1.091) | 0.020 (0.013) | 721.539^*^ (388.129) | 0.024^**^ (0.012) |
| Month of purchase:11/18 | 3.919^***^ (1.114) | 0.048^***^ (0.013) | 1180.990^***^ (392.811) | 0.038^***^ (0.012) |
| Month of purchase:12/18 | 20.098^***^ (1.174) | 0.222^***^ (0.013) | 4491.213^***^ (405.152) | 0.135^***^ (0.012) |
| Month of purchase:1/19 | 4.823^***^ (1.496) | 0.065^***^ (0.018) | 2437.554^***^ (546.269) | 0.083^***^ (0.017) |
| Month of purchase:2/19 | 6.022^***^ (1.518) | 0.080^***^ (0.018) | 2697.588^***^ (548.441) | 0.091^***^ (0.017) |
| Month of purchase:3/19 | 5.657^***^ (1.520) | 0.074^***^ (0.018) | 2493.771^***^ (552.152) | 0.083^***^ (0.017) |
| Month of purchase:4/19 | 8.484^***^ (1.547) | 0.108^***^ (0.018) | 3153.615^***^ (558.208) | 0.103^***^ (0.017) |
| Month of purchase:5/19 | 6.481^***^ (1.554) | 0.084^***^ (0.019) | 2754.023^***^ (569.824) | 0.090^***^ (0.018) |
| Scotland * Month of purchase:5/17 | 2.619 (1.598) | 0.029 (0.019) | 698.617 (572.087) | 0.021 (0.017) |
| Scotland * Month of purchase:6/17 | 3.287^**^ (1.591) | 0.036^*^ (0.019) | 290.935 (566.464) | 0.007 (0.017) |
| Scotland * Month of purchase:7/17 | 4.165^***^ (1.615) | 0.048^**^ (0.019) | 1143.185^**^ (574.821) | 0.034^*^ (0.018) |
| Scotland * Month of purchase:8/17 | 4.278^***^ (1.582) | 0.048^***^ (0.018) | 892.961 (575.767) | 0.026 (0.018) |
| Scotland * Month of purchase:9/17 | 2.485 (1.644) | 0.026 (0.019) | 410.516 (588.475) | 0.011 (0.018) |
| Scotland * Month of purchase:10/17 | 2.572 (1.632) | 0.029 (0.019) | 277.343 (586.196) | 0.008 (0.018) |
| Scotland * Month of purchase:11/17 | 1.611 (1.641) | 0.012 (0.019) | -99.619 (581.798) | -0.009 (0.018) |
| Scotland * Month of purchase:12/17 | 3.347^*^ (1.832) | 0.030 (0.020) | 827.623 (623.353) | 0.022 (0.019) |
| Scotland * Month of purchase:1/18 | 1.998 (1.600) | 0.021 (0.019) | 347.302 (585.039) | 0.008 (0.018) |
| Scotland * Month of purchase:2/18 | 1.726 (1.637) | 0.016 (0.019) | 306.535 (587.031) | 0.006 (0.018) |
| Scotland * Month of purchase:3/18 | 2.306 (1.630) | 0.025 (0.019) | 520.422 (583.349) | 0.015 (0.018) |
| Scotland * Month of purchase:4/18 | 2.079 (1.631) | 0.019 (0.019) | 385.977 (586.201) | 0.007 (0.018) |
| Scotland * Month of purchase:5/18 | 2.579 (1.616) | 0.028 (0.019) | 483.852 (585.469) | 0.014 (0.018) |
| Scotland * Month of purchase:6/18 | 1.802 (1.677) | 0.018 (0.020) | 382.164 (594.926) | 0.010 (0.018) |
| Scotland * Month of purchase:7/18 | 2.876^*^ (1.675) | 0.031 (0.020) | 604.597 (606.386) | 0.016 (0.019) |
| Scotland * Month of purchase:8/18 | 4.126^**^ (1.622) | 0.045^**^ (0.019) | 1110.317^*^ (588.004) | 0.031^*^ (0.018) |
| Scotland * Month of purchase:9/18 | 1.234 (1.691) | 0.005 (0.020) | 273.138 (606.099) | -0.001 (0.019) |
| Scotland * Month of purchase:10/18 | 2.383 (1.646) | 0.027 (0.019) | 366.952 (590.932) | 0.010 (0.018) |
| Scotland * Month of purchase:11/18 | 1.053 (1.697) | 0.008 (0.020) | -72.562 (607.097) | -0.007 (0.019) |
| Scotland * Month of purchase:12/18 | 1.901 (1.858) | 0.017 (0.021) | 637.328 (641.489) | 0.017 (0.019) |
| Scotland * Month of purchase:1/19 | 0.345 (1.646) | -0.002 (0.020) | -108.511 (600.521) | -0.008 (0.018) |
| Scotland * Month of purchase:2/19 | 0.860 (1.684) | 0.005 (0.020) | -276.255 (609.671) | -0.012 (0.019) |
| Scotland * Month of purchase:3/19 | 1.871 (1.659) | 0.018 (0.019) | -93.404 (601.064) | -0.007 (0.018) |
| Scotland * Month of purchase:4/19 | 1.216 (1.713) | 0.010 (0.020) | -110.224 (611.896) | -0.007 (0.019) |
| Scotland * Month of purchase:5/19 | 1.342 (1.801) | 0.010 (0.021) | -181.030 (656.583) | -0.008 (0.020) |
| Age of shopper | - | - | - | - |
| Age of shopper squared/100 | -3.767^***^ (0.523) | -0.050^***^ (0.007) | -1238.241^***^ (193.040) | -0.043^***^ (0.006) |
| Total people in household | 5.310^***^ (0.727) | 0.060^***^ (0.008) | 1926.599^***^ (248.566) | 0.056^***^ (0.007) |
| Children dummy | -4.704^**^ (1.942) | -0.052^**^ (0.023) | -1046.060 (790.715) | -0.029 (0.024) |
| Log years in panel | -4.262^***^ (0.801) | -0.050^***^ (0.009) | -1762.691^***^ (305.116) | -0.053^***^ (0.009) |
| Spend: Non-food | 1.370^***^ (0.037) | 0.010^***^ (0.000) | 478.257^***^ (12.736) | 0.010^***^ (0.000) |
| Observations | 687059 | 687059 | 687059 | 687059 |
| Households | 8051 | 8051 | 8051 | 8051 |
| Common trends test p-value | 0.071 | 0.106 | 0.354 | 0.432 |

Standard errors in parentheses ^*^ *p* < 0.10, ^**^ *p* < 0.05, ^***^ *p* < 0.01

Note: age of shopper dropped due to collinearity.

**Table A20. Test of Common Trends Assumption for Multiplicative Product Category Level Models**

|  | Spend |  | Volume |  |
| --- | --- | --- | --- | --- |
| Category: | Chi2 | p | Chi2 | p |
| Canned food | 0.27 | 0.603 | 0.73 | 0.394 |
| Convenience food | 1.87 | 0.172 | 0.03 | 0.854 |
| Rice and pasta | 0.49 | 0.482 | 1.61 | 0.204 |
| Dairy | 0.53 | 0.465 | 1.76 | 0.184 |
| Fish | 3.11* | 0.078 | 4.03** | 0.045 |
| Meat | 1.93 | 0.164 | 1.48 | 0.223 |
| Cereal | 0.33 | 0.567 | 0.26 | 0.609 |
| Fruit and veg | 1.23 | 0.268 | 1.37 | 0.241 |
| Tea and coffee | 4.13** | 0.042 | 2.15 | 0.142 |
| Juice | 0.23 | 0.634 | 0.23 | 0.634 |
| Home cooking | 0.48 | 0.489 | 0.60 | 0.438 |
| Biscuits and bakery | 1.67 | 0.196 | 1.26 | 0.262 |
| Crisps and snacks | 1.82 | 0.178 | 1.51 | 0.220 |
| Soft drinks | 1.58 | 0.209 | 2.04 | 0.154 |
| Confectionary | 0.30 | 0.583 | 0.12 | 0.729 |
| Slimming | 2.86* | 0.091 | 3.73* | 0.053 |

Category-level models include age of shopper, age of shopper squared/100, total people in household, children dummy, log years in panel, non-food spend in week, and month of purchase.

* p < 0.10, ** p < 0.05, *** p < 0.01

**Table A21. Test of Anticipation on Influence of MUP on Overall Household Food Purchases**

|  | Percent | Percent |
| --- | --- | --- |
|  | Spend | Volume |
| Post-MUP | 0.003 [-0.009,0.014] | 0.017^***^ [0.006,0.028] |
| Prior to MUP: 12-7 weeks | -0.001 [-0.013,0.012] | 0.007 [-0.005,0.019] |
| Prior to MUP: 6-1 weeks | -0.019^***^ [-0.032,-0.006] | -0.007 [-0.019,0.006] |
| Exposed to MUP in Scotland | -0.012^**^ [-0.022,-0.002] | -0.008 [-0.018,0.003] |
| Age of shopper | 0.052^***^ [0.034,0.070] | 0.041^***^ [0.023,0.058] |
| Age of shopper squared/100 | -0.050^***^ [-0.063,-0.037] | -0.043^***^ [-0.056,-0.031] |
| Total people in household | 0.059^***^ [0.044,0.075] | 0.056^***^ [0.042,0.070] |
| Children dummy | -0.050^**^ [-0.096,-0.005] | -0.029 [-0.076,0.019] |
| Log years in panel | -0.050^***^ [-0.069,-0.032] | -0.053^***^ [-0.070,-0.035] |
| Spend: Non-food | 0.010^***^ [0.010,0.011] | 0.010^***^ [0.009,0.011] |
| Month of purchase: February | 0.023^***^ [0.015,0.030] | 0.012^***^ [0.005,0.019] |
| Month of purchase: March | 0.043^***^ [0.036,0.051] | 0.020^***^ [0.013,0.026] |
| Month of purchase: April | 0.035^***^ [0.027,0.042] | 0.018^***^ [0.011,0.025] |
| Month of purchase: May | 0.026^***^ [0.015,0.037] | 0.011^**^ [0.001,0.021] |
| Month of purchase: June | 0.017^***^ [0.004,0.029] | 0.002 [-0.009,0.013] |
| Month of purchase: July | 0.012^*^ [-0.000,0.025] | 0.005 [-0.007,0.016] |
| Month of purchase: August | 0.022^***^ [0.009,0.034] | 0.007 [-0.004,0.018] |
| Month of purchase: September | 0.017^**^ [0.004,0.030] | -0.007 [-0.019,0.005] |
| Month of purchase: October | 0.025^***^ [0.012,0.038] | -0.009 [-0.020,0.003] |
| Month of purchase: November | 0.046^***^ [0.033,0.059] | -0.000 [-0.012,0.011] |
| Month of purchase: December | 0.223^***^ [0.209,0.238] | 0.108^***^ [0.095,0.121] |
| Observations | 687059 | 687059 |
| Households | 8051 | 8051 |
| Pseudo-R^2^ | 0.407 | 0.487 |

95% confidence intervals in brackets * p < 0.10, ** p < 0.05, *** p < 0.01

**Table A22. Effects of Anticipation (alternative implementation date) on Influence of MUP on Overall Household Food Purchases**

|  | Percent | Percent |
| --- | --- | --- |
|  | Spend | Volume |
| Post-MUP | -0.007 [-0.015,0.002] | 0.004 [-0.004,0.012] |
| Exposed to MUP in Scotland | -0.011^**^ [-0.021,-0.002] | -0.008^*^ [-0.018,0.001] |
| Age of shopper | 0.060^***^ [0.044,0.076] | 0.052^***^ [0.036,0.068] |
| Age of shopper squared/100 | -0.050^***^ [-0.063,-0.037] | -0.043^***^ [-0.055,-0.031] |
| Total people in household | 0.059^***^ [0.044,0.075] | 0.056^***^ [0.042,0.070] |
| Children dummy | -0.050^**^ [-0.096,-0.005] | -0.029 [-0.077,0.019] |
| Log years in panel | -0.050^***^ [-0.068,-0.032] | -0.052^***^ [-0.070,-0.035] |
| Spend: Non-food | 0.010^***^ [0.010,0.011] | 0.010^***^ [0.009,0.011] |
| Month of purchase: February | 0.022^***^ [0.015,0.030] | 0.014^***^ [0.007,0.020] |
| Month of purchase: March | 0.044^***^ [0.036,0.052] | 0.020^***^ [0.013,0.027] |
| Month of purchase: April | 0.037^***^ [0.028,0.045] | 0.017^***^ [0.009,0.025] |
| Month of purchase: May | 0.033^***^ [0.023,0.042] | 0.021^***^ [0.012,0.029] |
| Month of purchase: June | 0.025^***^ [0.015,0.035] | 0.013^***^ [0.004,0.023] |
| Month of purchase: July | 0.020^***^ [0.010,0.030] | 0.016^***^ [0.007,0.026] |
| Month of purchase: August | 0.030^***^ [0.020,0.040] | 0.019^***^ [0.010,0.028] |
| Month of purchase: September | 0.025^***^ [0.014,0.036] | 0.005 [-0.005,0.015] |
| Month of purchase: October | 0.033^***^ [0.022,0.043] | 0.003 [-0.006,0.012] |
| Month of purchase: November | 0.054^***^ [0.043,0.065] | 0.011^**^ [0.001,0.021] |
| Month of purchase: December | 0.231^***^ [0.219,0.243] | 0.120^***^ [0.109,0.130] |
| Observations | 687059 | 687059 |
| Households | 8051 | 8051 |
| Pseudo-R^2^ | 0.407 | 0.487 |

95% confidence intervals in brackets * p < 0.10, ** p < 0.05, *** p < 0.01
